# Doctor-diagnosed health problems in a region with a high density of concentrated animal feeding operations: a cross-sectional study

**DOI:** 10.1186/s12940-016-0123-2

**Published:** 2016-02-17

**Authors:** Mariëtte Hooiveld, Lidwien A. M. Smit, Femke van der Sman-de Beer, Inge M. Wouters, Christel E. van Dijk, Peter Spreeuwenberg, Dick J. J. Heederik, C. Joris Yzermans

**Affiliations:** NIVEL, Netherlands institute for health services research, P.O. Box 1568, 3500BN Utrecht, The Netherlands; Institute for Risk Assessment Sciences, Division Environmental Epidemiology, Utrecht University, Utrecht, The Netherlands

**Keywords:** Livestock, Poultry, Public health, Environmental exposure, Multilevel analysis

## Abstract

**Background:**

There is growing interest in health risks of residents living near concentrated animal feeding operations (CAFOs). Previous research mostly focused on swine CAFOs and self-reported respiratory conditions. The aim was to study the association between the presence of swine, poultry, cattle and goat CAFOs and health of Dutch neighbouring residents using electronic medical records from general practitioners (GPs).

**Methods:**

Data for the year 2009 were collected of 119,036 inhabitants of a rural region with a high density of CAFOs using information from GIAB (high exposed population). A comparison was made with GP data from 78,060 inhabitants of rural areas with low densities of CAFOs (low exposed population). Associations between the number of CAFOs near residents’ homes and morbidity were determined by multilevel (cross-classified) logistic regression.

**Results:**

In 2009, the prevalence of most respiratory and gastrointestinal conditions was similar in the high and low exposed population. Exceptions were pneumonia, atopic eczema and unspecified infectious diseases with an increased prevalence, and sinusitis with a decreased prevalence in the high exposed population. Within the high CAFO density region, the number of poultry, cattle and swine CAFOs near residents’ homes was not associated with allergic, respiratory or gastrointestinal conditions. Conversely, each additional goat CAFO within the postal code area of residents’ homes significantly increased the odds of unspecified infectious disease and pneumonia by 87 and 41 percent, respectively.

**Conclusions:**

Using GP records, pneumonia and unspecified infectious diseases were positively associated with the number of goat CAFOs near residents’ homes, but no association was found between swine, cattle, and poultry CAFOs and respiratory, allergic or gastrointestinal conditions.

**Electronic supplementary material:**

The online version of this article (doi:10.1186/s12940-016-0123-2) contains supplementary material, which is available to authorized users.

## Background

There is growing attention for health problems of residents living in the proximity of livestock farms. Radon et al. suggested that a high density of animal houses in the proximity of neighbouring residents adversely affects respiratory health [1]. In 2010, a systematic review described the association between livestock farms and measures of respiratory, gastrointestinal (GI) and mental health of individuals living near these facilities [2]. The authors concluded that there was little compelling evidence for a consistent strong association between proximity to animal farms and clinical measures of disease. More recent studies among neighbouring residents showed either no, or inverse, associations with respiratory health and allergies or positive associations with physical symptoms [3–7].

Although several studies have addressed the possible adverse health effects of animal farm emissions among neighbouring residents, there are still issues that need further investigation. First, in several studies it was unclear whether the research involved animal farms in general or more specifically concentrated animal feeding operations (CAFOs), because detailed information about the livestock population size was lacking [2]. Second, most research has been performed on swine CAFOs specifically. Third, most published studies are based on patient-reported symptoms, while it is known that prevalence estimates derived from self-reported data deviate from those obtained from general practitioners’ (GPs) medical records [8]. Finally, most studies focussed on respiratory health and allergies, but only a few addressed the possible relation between CAFOs and GI outcomes [9, 10].

In the Netherlands, the number of CAFOs (also called mega-farms) has doubled between the years 2000 and 2009 [11]. In contrast to other countries, in the Netherlands a high animal density is paralleled by a high human population density, resulting in a large human population potentially at risk of adverse health effects. After an outbreak of swine fever in the Netherlands in 1997, the government decided to allocate specific areas for intensive livestock farming. The eastern part of the province Noord-Brabant and the northern part of the adjacent province of Limburg, also known as ‘the Peel’, is a region with mainly poor, sandy soils, and a history of livestock farming. Within this Peel region, many areas were allocated for intensive livestock farming. After the Q fever epidemic in 2009, there were growing concerns about adverse health effects of living near CAFOs in general, especially in this Peel region. In response to this, the Dutch government decided to fund the described study on the health of residents. We carried out a study evaluating health problems of more than 100,000 individuals living in this rural region with a high density of farm animals, using data obtained from electronic medical records (EMRs) registered by Dutch GPs [12]. Previously, we reported the results of associations between the presence and numbers of farm animals within 5 km of the home address and GP-registered health problems [5, 13]. However, reports about Q fever outbreaks suggested windborne spread of *Coxiella burnetii* aerosols over several kilometres [14]. In addition, airborne cow allergens have been found at distances up to 4.8 km of dairy facilities [15]. On the other hand, for a given number of animals in the home environment, potential health effect might be different with many small-scale farms compared to one large farm. Therefore, the aim of the current study was to evaluate associations of swine, poultry, cattle and goat CAFOs with health problems among neighbouring residents using a semi-individual design. We first compared the prevalence of GP-registered health problems of residents living in the rural region with a high CAFO density to those living in rural regions with a low CAFO density. Next, within the high CAFO density region, we evaluated health effects with the number of CAFOs located in the proximity of residents’ homes.

## Methods

### Study population

All Dutch citizens are obligatory registered with a general practice in the proximity of their home (usually 10 km) and GPs have a gatekeeper role for access to specialized, secondary care. Therefore, records kept by GPs provide a good and complete picture of people’s health, while the number of enlisted patients can be used a the denominator in epidemiological studies. The guidelines of the Dutch College of General Practitioners require that GPs record morbidity data of their patients in EMRs using the International Classification of Primary Care (ICPC) [16, 17].

**Fig. 1:**
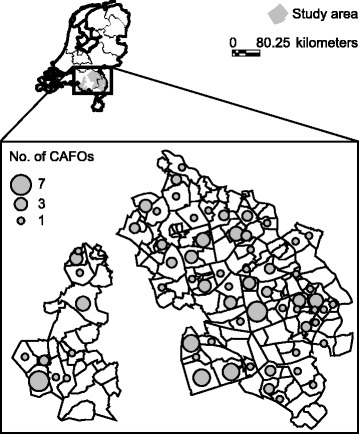
High CAFO density study region: eastern part of province of Noord-Brabant and northern part of province of Limburg. For each postal code area the number of CAFOs is indicated (GIAB 2009, Alterra Wageningen UR, the Netherlands)

The study region was chosen, as it is the part of the Netherlands with the highest density of CAFOs. No other regions in the Netherlands have this many CAFOs. The region covered the eastern part of the province Noord-Brabant, excluding the urban areas, and the north-western part of the province Limburg (Fig. 1). These two provinces, out of a total of twelve, have 208 (48 %) of all CAFOs in the Netherlands [11]. Between December 2009 and March 2010, general practices located in rural postal code areas (<1500 addresses per km^2^ according to National Statistics) in this region with high CAFO density were asked to participate through regional professional newsletters. Forty-nine practices applied, whereof 28 (57 %) practices that met pre-defined registration quality criteria were included in the study [13]. Fourteen practices included were participants of NIVEL Primary Care Database, formerly known as Netherlands Information Network of General Practice (LINH) [18]. EMR data of the year 2009 was available for 119,036 enlisted persons (19 % of the total population in this region), including those who did not consult their GP. We refer to this population as the high exposed group.

A comparison was made with patients of general practices of NIVEL Primary Care Database located in other rural postal code areas throughout the Netherlands. Using the same quality criteria, we included 78,060 enlisted patients of 22 eligible general practices. We refer to this population as low exposed to CAFO.

The study was carried out according to Dutch legislation on privacy and the Dutch ‘Code of Conduct for the use of data in health research’ [19]. The study did not fall within the scope of the Dutch Medical Research Involving Human Subjects Act and therefore did not require ethical approval.

### Data collection

Year of birth, gender, and postal code area (4-digits) of patients’ home address were abstracted from the EMRs of all enlisted patients in the year 2009. For each patient, morbidity data were derived from all consultations and prescriptions recorded in the EMRs. Consultations concerning the same health problem were clustered into episodes of care, defined as all encounters for the management of the same specific health problem, using the computerized algorithm EPICON [20].

Rates were calculated as the total number of episodes of enlisted patients divided by the total number of enlisted patients. One-year prevalence rates were calculated for outcome variables chosen on results from previously published studies: atopic diseases, respiratory and GI infections and ‘other infectious disease’ which is used for the registration of Q fever [13]. For several chronic disorders among the selected outcomes, also three-year prevalence rate (2007–2009) were calculated, as some patients with a chronic disorder do not visit a GP yearly and would not be included in the yearly prevalence rates.

Data on the background of the patient (Dutch or foreign) and standardized household income, a proxy for socioeconomic status (SES), in the year 2009 were available from Statistics Netherlands for 84.5 % and 85.2 % of the patients in the high and low exposed populations, respectively.

### Exposure assessment

Numbers and type of CAFOs located in the postal code areas for the year 2009 were obtained from the Dutch Agricultural Geographic Information System GIAB (GIAB 2009, Alterra Wageningen UR, the Netherlands). CAFOs were defined as facilities with >250 dairy cows, >2500 veal calves, >7500 finishing pigs, >1200 breeding sows, >120,000 laying hens, >220,000 broilers, or >1500 goats [21]. The number of animals kept in the CAFOs were comparable to the US Environmental Protection Agency (EPA) definition of a medium or large CAFO [22]. Data on CAFOs were only available at the postal code area level, to protect the privacy of the farmers.

### Statistical analyses

#### Comparison of prevalence rate between high and low exposed groups

Multilevel logistic regression analyses with two-level hierarchical structured data (i.e. patients being clustered within general practices) were performed with MLwiN 2.02. [23], adjusting for age (polynomial), gender and registry duration. The centred values of these independent variables (i.e. 40 years of age, 49.3 % males and full year registry duration) were used to obtain comparable estimates for the high and low exposed populations. The potential confounding effect by SES was evaluated by including standardized household income in the models. However, since this did not alter the results, only results without adjustment for SES are shown.

Since prevalence rates of several studied conditions are age-related [13], additional multilevel analyses were performed for specific age groups. Gastroenteritis presumed infection, acute upper respiratory infection, acute laryngitis/tracheitis, asthma and atopic eczema were evaluated among the very young children aged 0 to 4 years. In addition, rates of children aged 0 to 14 years were evaluated for gastroenteritis presumed infection, asthma and atopic eczema. COPD was evaluated for adults aged 45 years or older and for the elderly aged 60 years or more. Results are presented as odds ratios (ORs) with 95 % confidence intervals (CIs).

#### Semi-individual analyses

Morbidity data of the year 2009 of the high exposed population were used to study the possible association between prevalence rates of above mentioned conditions and the number of CAFOs located in the proximity (postal code area) of the patients’ home. We determined the association with the total number of CAFOs (irrespective of the type of animals kept), as well as specific numbers of swine, poultry, cattle and goat CAFOs.

Multilevel logistic regression analysis was used with individual patients (level 1) nested within general practices (level 2). CAFO data were available at the postal code level only. Since patients were classified both by their general practice and by the postal code area of their homes, postal code areas of the patients’ home addresses were included in the models as separate level (cross-classified) [24].

We used two different models. The first evaluated the effects of the number of CAFOs located in the same postal code of the patients’ home address. However, since people move around in their living environment, the second model evaluated the indirect effects of CAFOs located in the adjacent postal code areas, while ignoring any CAFOs in the residential postal code. Models were performed with MLwiN 2.02. [23], adjusting for age (polynomial), gender, registry duration, the number of inhabitants in the postal code area and total surface area.

## Results

Table 1 shows the descriptives of the high and low CAFO density regions and the characteristics of both populations. In 2009, 129 (30 %) out of 430 CAFOs registered in the Netherlands were located in the high CAFO density region. The 129 CAFOs were located in 66 of the 171 postal code areas (Fig. 1). Forty-four percent (*n* = 52,773) of the population in this region was living in a postal code area with one of more CAFOs. The mean surface area of these postal codes was 10 km^2^, ranging from 1.0 km^2^ to 48.5 km^2^.

**Table 1:** Characteristics of the high and low CAFO density regions and characteristics of the general practitioners’ patients populations, 2009

	High CAFO density region	Low CAFO density regions
Characteristics of the regions		
No. of postal code areas	171	785
No. of CAFOs ^a^	129	10
Swine	85	2
Poultry	28	1
Cattle	10	7
Goat	6	0
No. of general practices	28	22
No. of general practitioners	66	32
Characteristics of the populations		
Total No. of patients ^b^	119,036	78,060
Gender (females); n (%)	58,952 (49.5)	38,983 (49.9)
Age (yrs); mean ± SD	40 ± 23	39 ± 23
No. of patients aged 0–14 yrs; n (%)	21,509 (18.1)	14,805 (19.0)
No. of patients aged ≥65 yrs; n (%)	18,318 (15.4)	11,880 (15.2)
Standardized household income x € 1000 ^c^; mean ± SD	24.6 ± 15.5	24.3 ± 15.6

Note. *CAFO* concentrated animal feeding operation, *SD* standard deviation

^a^Data from GIAB (GIAB 2009, Alterra Wageningen UR, the Netherlands)

^b^All enlisted patients in the year 2009

^c^Calculations made by NIVEL using custom-made microdatasets from Statistics Netherlands concerning NIVEL project 7093

The low CAFO density regions consisted of 785 postal code areas with a total of 10 CAFOs distributed over 7 postal code areas. Three percent (*n* = 2384) of the low exposed population resided in these 7 postal code areas. Mean surface of the low exposed postal code areas was 11.1 km^2^ (sd 10.6).

Both populations were comparable with respect to standardized household income, place of birth (>90 % in the Netherlands), and marital status (not in table). The average of the standardized household income of both populations was comparable to that of the Dutch population in the year 2009 (i.e. € 23,300) [25].

Table 2 shows one-year prevalence rates of the populations in the high and low CAFO density regions. In general, the prevalence of respiratory and GI diseases in both regions was comparable. Exceptions were ‘other infectious disease’ (including Q fever), pneumonia and atopic eczema with an increased prevalence, and sinusitis with a decreased prevalence in the high exposed population.

**Table 2:** One-year prevalence rates (per 1000 patients) and odds ratios of the general practitioners’ patients populations in regions with high and low CAFO density, 2009

Diagnosis	High CAFO density region	Low CAFO density regions^a^	OR (95 % CI)^b^
Other infectious disease	3.32	1.71	1.95 (1.17-3.26)
Gastrointestinal infection	0.81	0.75	1.08 (0.65-1.80)
Gastroenteritis presumed infection	4.36	4.99	0.87 (0.62-1.22)
Age group 0–4 yrs	44.72	54.22	0.82 (0.58-1.14)
Age group 0–14 yrs	10.77	13.16	0.82 (0.58-1.15)
Chronic enteritis	3.29	2.47	1.33 (0.99-1.79)
Allergic conjunctivitis	3.64	4.59	0.79 (0.57-1.09)
Acute URI	27.59	28.41	0.97 (0.73-1.29)
Age group 0–4 yrs	177.77	182.63	0.97 (0.73-1.29)
Sinusitis acute/chronic	25.56	37.14	0.68 (0.52-0.88)
Laryngitis/tracheitis acute	0.75	1.01	0.74 (0.40-1.39)
Age group 0–4 yrs	11.57	16.42	0.70 (0.43-1.14)
Influenza	8.48	9.72	0.87 (0.53-1.42)
Pneumonia	5.58	3.93	1.42 (1.12-1.82)
Asthma	24.69	26.11	0.94 (0.73-1.22)
Age group 0–4 yrs	28.49	21.80	1.32 (0.89-1.95)
Age group 0–14 yrs	24.74	23.27	1.06 (0.81-1.41)
Age group ≥ 65 yrs	19.25	20.49	0.94 (0.65-1.36)
Hay fever	30.21	37.25	0.81 (0.65-1.00)
COPD	3.09	3.12	0.99 (0.78-1.26)
Age group ≥ 45 yrs	27.83	28.48	0.98 (0.77-1.24)
Age group ≥ 60 yrs	51.54	53.55	0.96 (0.75-1.23)
Atopic eczema	7.75	5.68	1.37 (1.05-1.79)
Age group 0–4 yrs	77.65	60.89	1.30 (1.02-1.65)
Age group 0–14 yrs	25.79	21.22	1.22 (0.96-1.55)

Note. *OR* odds ratio, *CI* confidence interval, *URI* upper respiratory infection, *COPD* chronic obstructive pulmonary disease

^a^Reference group

^b^Adjusted for age, gender, and registry duration

For several chronic disorders, three-year prevalence rates were estimated (Table 3). Three-year prevalences of the studied chronic disorders were comparable in both populations, with the exception of atopic eczema in children aged 0 to 4 years. Atopic eczema was more likely to occur in the high CAFO density region compared to the low exposed regions, both for the one-year and three-years prevalence.

**Table 3:** Three-year prevalence rates (per 1000 patients) and odds ratios of the general practitioners’ patients populations in high and low CAFO density regions, 2007-2009

Diagnosis	High CAFO density region	Low CAFO density regions^a^	OR (95 % CI)^b^
Chronic enteritis	4.72	4.35	1.09 (0.83-1.42)
Asthma	44.32	56.16	0.78 (0.58-1.05)
Age group 0–4 yrs	60.39	49.69	1.23 (0.83-1.83)
Age group 0–14 yrs	49.56	54.20	0.91 (0.69-1.20)
COPD	6.40	7.84	0.82 (0.63-1.06)
Age group ≥ 45 yrs	40.66	49.72	0.81 (0.61-1.08)
Age group ≥ 60 yrs	73.63	90.91	0.79 (0.60-1.06)
Atopic eczema	16.73	13.92	1.21 (0.93-1.57)
Age group 0–4 yrs	233.04	190.27	1.29 (1.08-1.55)
Age group 0–14 yrs	63.47	56.24	1.14 (0.93-1.39)

Note. *OR* odds ratio, *CI* confidence interval, *COPD* chronic obstructive pulmonary disease

^a^Reference group

^b^Adjusted for age, gender, and registry duration

Data from the high CAFO density region were used to determine the relation between the diagnoses presented in Tables 2 and 3 and the number of CAFOs in the proximity of the patients’ homes. Table 4 shows the results for all types of CAFOs, as well as animal-specific CAFOs with the most pronounced associations. In Additional file 1, all results of the analyses are shown.

**Table 4:** Association between the number of CAFOs in postal code areas and health problems within the high CAFO density region, 2009

		OR (95 % CI)^a^ for each additional CAFO	
Diagnosis	Type of CAFO^b^	Within the postal code area	In adjacent postal code areas
Other infectious disease	All	0.88 (0.75-1.03)	1.00 (0.95-1.05)
	Goat only	1.87 (1.11-3.13)	1.77 (1.30-2.41)
Gastroenteritis presumed infection	All	0.93 (0.86-1.01)	1.01 (0.99-1.04)
Chronic enteritis	All	0.99 (0.85-1.15)	0.97 (0.93-1.01)
	Poultry only	1.13 (0.79-1.62)	0.89 (0.79-1.00)
Allergic conjunctivitis	All	1.00 (0.88-1.14)	1.02 (0.98-1.06)
	Goat only	1.57 (0.99-2.50)	1.42 (1.10-1.83)
Acute URI	All	1.03 (0.96-1.11)	1.03 (1.01-1.06)
	Swine only	1.10 (0.98-1.24)	1.04 (1.01-1.08)
	Goat only	0.95 (0.69-1.29)	1.37 (1.12-1.66)
Pneumonia	All	1.03 (0.95-1.11)	1.00 (0.98-1.03)
	Goat only	1.41 (1.08-1.84)	1.17 (1.00-1.37)
Asthma	All	1.03 (0.98-1.08)	1.00 (0.98-1.01)
	Goat only	1.16 (0.94-1.43)	1.15 (1.01-1.31)
Age group 0–4 yrs	All	0.82 (0.66-1.02)	1.02 (0.96-1.08)
Age group 0–4 yrs	Goat only	0.92 (0.45-1.88)	1.62 (1.15-2.28)
Age group 0–14 yrs	All	0.97 (0.87-1.08)	0.99 (0.97-1.03)
Age group 0–14 yrs	Goat only	1.40 (0.96-2.04)	1.37 (1.13-1.67)
Hay fever	All	1.01 (0.94-1.07)	1.00 (0.98-1.02)
	Goat only	1.19 (0.90-1.56)	1.22 (1.05-1.41)
COPD	All	1.04 (0.97-1.12)	1.00 (0.98-1.02)
Age group 45 yrs and older	All	1.07 (1.01-1.14)	1.00 (0.98-1.02)
Age group 60 yrs and older	All	1.13 (1.02-1.24)	0.98 (0.95-1.00)
Atopic eczema	All	1.02 (0.97-1.08)	1.01 (0.99-1.03)
	Goat only	1.26 (1.00-1.59)	1.19 (1.03-1.38)

Note. *CAFO* concentrated animal feeding operations, *OR* odds ratio, *CI* confidence interval, *URI* upper respiratory infection. See also Additional file 1

^a^Adjusted for age, gender, registry duration, the number of inhabitants in the postal code area and total surface area

^b^Defined as >250 dairy cows or >2500 veal calves (cattle CAFO), >7500 finishing pigs or >1200 breeding sows (swine CAFO), >120,000 laying hens or >220,000 broilers (poultry CAFO), or >1500 goats (goat CAFO)

Goat CAFOs showed the most statistically significant associations (Table 4). Patients with one additional goat CAFO in their residential postal code area were 1.41 times more likely to have pneumonia. In addition, the OR for pneumonia was 1.17 for patients with one additional goat CAFO in any of the adjacent areas. Since the two effects are additive, the odds of pneumonia was increased by 65 % (1.41*1.17) for patients living in a postal code area with one goat CAFO and an additional one in the adjacent areas. The presence of one additional goat CAFO inside the postal code area of patients’ homes significantly increased the odds of ‘other infectious diseases’ by 87 %, whereas each additional goat CAFO in any of the adjacent areas increased the odds by 77 %. In addition, goat CAFOs in adjacent postal code areas were positively associated with allergic conjunctivitis, asthma, hay fever, acute upper respiratory infection and atopic eczema. The presence of one additional goat CAFO within the postal code area of patients’ homes was positively associated with atopic eczema. For all types of CAFOs and for swine CAFOs in adjacent areas,positive associations were observed with acute upper respiratory infections. COPD among adult patients aged 45 years or older was positively associated with number of CAFOs in the postal code area of patients’ home. The association was stronger among the age group of 65 years or older.

## Discussion

The prevalence of respiratory and GI conditions in a Dutch rural region with a high CAFO density was comparable to other rural regions with a low CAFO density. Exceptions were pneumonia, atopic eczema and ‘other infectious diseases’ (among which Q fever) with a significantly higher prevalence. In the high CAFO density region, the number of goat CAFOs was positively associated with pneumonia and ‘other infectious diseases’. No relationship between the number of CAFOs and GI conditions was found.

The current study is part of a larger Dutch study in which the health effects of livestock farming among neighbouring residents were investigated during the years 2006 to 2009 [12]. Previously published results used individual-level density of all animal farms, irrespective of their size, within a 5 km radius around residents’ homes using a Geographic Information System [5, 13]. The present semi-individual study focussed more specifically on the number of CAFOs in or around the postal code area of residents’ homes, because potential health effects due to the presence of ‘mega farms’ are a growing topic of debate in the Netherlands. Despite these differences in design, both studies showed a positive association between goats and pneumonia as well as ‘other infectious diseases’(Q fever).

Between 2007 and 2009, a large Q fever outbreak took place in the high CAFO density region [26]. Q fever is caused by the bacterium *Coxiella burnetii* and humans become infected through inhalation of contaminated dust and aerosols, released by infected ruminants. The high prevalence of pneumonia found in our study coincided with the Q fever outbreak. However, additional evaluations of GP morbidity data of the year 2006 showed that the prevalence of pneumonia was 3.4 per 1000 persons in the high CAFO density region versus 2.5 per 1000 in the low CAFO density regions (OR = 1.37; 95 % CI = 0.89-2.09). Although not statistically different, this difference suggests that pneumonia prevalence rates in the study area were already increased in the year 2006, preceding the Q fever outbreak. In addition to goat CAFOs, the results from our individual-level study indicated that also smaller goat farms as well as poultry farms were associated with the increase of pneumonia cases in the study area [13].

In pre-school children, atopic eczema was more likely to occur in the proximity of goat CAFOs. It is unclear which farm exposures could be responsible for this finding. In contrast, numerous studies reported a reduced risk of allergic conditions such as atopic eczema, allergic rhinitis and allergic asthma among children who grew up on farms (reviewed in [27, 28]). In our previous individual-level study, we also found inverse associations between farm exposures and asthma and allergic rhinitis both in children (0–17 years) and adults [5].

COPD was more likely to occur in the close proximity of CAFOs. This is in contrary to our previous individual-level study, in which we found an inverse association between farm exposures and COPD [5]. This inverse association was counterintuitive, since it is not biologically plausible that farm-related exposure may protect against COPD. In addition, previous research showed that livestock farmers are at increased risk of developing COPD due to high levels of endotoxin and other microbial components in stable dust [29].

The present evidence on effects of CAFOs on respiratory, atopic or GI conditions is mostly limited to swine CAFOs [3, 6, 30–32]. We were able to study the separate effects of swine, cattle, poultry, and goat CAFOs. In contrast to the earlier studies, we observed only weak associations between swine CAFOs and health in neighbouring residents. However, results are difficult to compare, because none of the cited studies used information obtained from EMRs registered by GPs. Furthermore, most researchers used the traditional individual-level study design [3, 6, 27], while the current study can be considered as a semi-individual study [33]. Likewise to our study, Mirabelli et al. used a multilevel approach to study respiratory outcomes in public schools with children aged 12 to 14 years [31]. An increased risk of self-reported physician-diagnosed asthma was found in children with self-reported allergies who attended schools located within 3 miles distance of a swine CAFO, but there was no clear dose-response relationship.

In contrast to two ecological studies using hospital data [9, 10], we did not find a clear association between CAFOs and GI illness. In our study the prevalence of GI infections may be underestimated, because only people with severe illness will consult their GP. A community-based study using questionnaire data also captured less severe cases of GI illness [34]. Interestingly, they found intensive farming activities to be negatively associated with GI illness.

Our study has several strengths. Outcome variables were obtained from EMRs registered by GPs, which rules out the possibility of recall bias. In the Netherlands, the patient population of a GP can be used as the denominator in epidemiological studies. Exposure data were not self-reported; detailed CAFO data were available at the postal code level. We used multilevel cross-classified models to take into account that patients were classified both by their general practice and by their postal code area [24]. In contrast to individual-level and ecological studies, multilevel studies are able to simultaneously examine the role of individual- and group-level factors in the risk of disease [35]. Finally, compared to other studies we were able to use a study design with a control group, by analysing data from persons living in rural areas with few CAFOs.

Several factors may have influenced the internal validity of our study findings. First, we performed a cross-sectional analysis. Consequently, selection bias might have occurred. It is possible that subjects with incipient health problems possibly related to CAFOs have moved from the high exposed region before they could be included in our study. This would lead to an underestimation of effect estimates. Second, only limited information about possible confounders was available in the EMRs. We could adjust for registry duration, age, gender and SES, and at the postal code area level, additional adjustments were made for the number of inhabitants and total surface area, but we were unable to adjust for air pollution caused by industries or traffic. However, by excluding the urban areas (more than 1500 addresses per km^2^) we have reduced the influence of these other sources of air pollution.

## Conclusions

In conclusion, using GP medical records, we found positive associations between the number of goat CAFOs near residents’ homes and pneumonia as well as ‘other infectious disease’. No association was found between the number of CAFOs and GI conditions. Living nearby cattle, poultry or swine CAFOs was not related to a higher odds of respiratory or GI conditions.

### Ethical approval

The study was carried out according to Dutch legislation on privacy and the Dutch ‘Code of Conduct for the use of data in health research’. The study did not fall within the scope of the Dutch Medical Research Involving Human Subjects Act and therefore did not require ethical approval.

## Additional file

Additional file 1:
**Detailed results of association between the number of CAFOs in the high CAFO density region, 2009.** (DOCX 31 kb)

## Abbreviations

CAFO: concentrated animal feeding operation
GP: general practitioner
EMR: electronic medical record
ICPC: international classification of primary care
