# Supplementary material for: Doctor-diagnosed health problems in a region with a high density of concentrated animal feeding operations: a cross-sectional study
Source: Environ Health. 2016 Feb 17;15:24. doi: 10.1186/s12940-016-0123-2 (PMC4758110; doi:10.1186/s12940-016-0123-2)
Supplement: Additional file 1: — Detailed results of association between the number of CAFOs in the high CAFO density region, 2009. (DOCX 31 kb) [file 12940_2016_123_MOESM1_ESM.docx]

**Appendix I:** Detailed results of association between the number of CAFOs in the high CAFO density region, 2009

|  |  | Within the postal code area | | | | |  | | In adjacent postal code areas | | | | | |
| --- | --- | --- | --- | --- | --- | --- | --- | --- | --- | --- | --- | --- | --- | --- |
| Diagnosis | Type of CAFO^b^ | non-exposed cases | exposed cases | regression coefficient | standard error | OR (95% CI)^a^ | |  | | non-exposed cases | exposed cases | regression coefficient | standard error | OR (95% CI)^a^ |
| Other infectious | All | 163 | 446 | -0.129 | 0.082 | 0.88 (0.75-1.03) | |  | | 26 | 583 | -0.001 | 0.025 | 1.00 (0.95-1.05) |
| disease | Cattle only | 583 | 26 | -0.042 | 0.218 | 0.96 (0.63-1.47) | |  | | 393 | 216 | 0.083 | 0.150 | 1.09 (0.81-1.46) |
|  | Goat only | 423 | 186 | 0.624 | 0.264 | 1.87 (1.11-3.13) | |  | | 224 | 385 | 0.570 | 0.158 | 1.77 (1.30-2.41) |
|  | Poultry only | 580 | 29 | -0.330 | 0.247 | 0.72 (0.44-1.17) | |  | | 282 | 327 | -0.082 | 0.073 | 0.92 (0.80-1.06) |
|  | Swine only | 346 | 263 | -0.210 | 0.126 | 0.81 (0.63-1.04) | |  | | 42 | 567 | -0.010 | 0.036 | 0.99 (0.92-1.06) |
| Gastroenteritis | All | 655 | 517 | -0.072 | 0.041 | 0.93 (0.86-1.01) | |  | | 123 | 1049 | 0.013 | 0.013 | 1.01 (0.99-1.04) |
| presumed infection | Cattle only | 1119 | 53 | -0.074 | 0.115 | 0.93 (0.74-1.16) | |  | | 777 | 395 | 0.100 | 0.075 | 1.10 (0.95-1.28) |
|  | Goat only | 1001 | 171 | 0.246 | 0.172 | 1.28 (0.91-1.79) | |  | | 914 | 258 | -0.098 | 0.104 | 0.91 (0.74-1.11) |
|  | Poultry only | 1078 | 94 | -0.216 | 0.111 | 0.81 (0.65-1.00) | |  | | 496 | 676 | -0.008 | 0.036 | 0.99 (0.92-1.07) |
|  | Swine only | 806 | 366 | -0.082 | 0.064 | 0.92 (0.81-1.04) | |  | | 157 | 1015 | 0.035 | 0.019 | 1.04 (1.00-1.07) |
| Chronic enteritis | All | 137 | 124 | -0.011 | 0,076 | 0.99 (0.85-1.15) | |  | | 42 | 219 | -0.029 | 0.020 | 0.97 (0.93-1.01) |
|  | Cattle only | 245 | 16 | 0.020 | 0.179 | 1.02 (0.72-1.45) | |  | | 194 | 67 | -0.183 | 0.118 | 0.83 (0.66-1.05) |
|  | Goat only | 225 | 36 | 0.082 | 0.281 | 1.09 (0.63-1.88) | |  | | 195 | 66 | -0.027 | 0.149 | 0.97 (0.73-1.30) |
|  | Poultry only | 237 | 24 | 0.124 | 0.183 | 1.13 (0.79-1.62) | |  | | 115 | 146 | -0.117 | 0.059 | 0.89 (0.79-1.00) |
|  | Swine only | 178 | 83 | -0.100 | 0.130 | 0.90 (0.70-1.16) | |  | | 53 | 208 | -0.015 | 0.031 | 0.98 (0.93-1.05) |
| Allergic | All | 232 | 212 | 0.001 | 0.066 | 1.00 (0.88-1.14) | |  | | 58 | 386 | 0.019 | 0.019 | 1.02 (0.98-1.06) |
| conjunctivitis | Cattle only | 431 | 13 | 0.029 | 0.182 | 1.03 (0.72-1.47) | |  | | 305 | 139 | 0.026 | 0.110 | 1.03 (0.83-1.27) |
|  | Goat only | 370 | 74 | 0.452 | 0.236 | 1.57 (0.99-2.50) | |  | | 294 | 150 | 0.352 | 0.129 | 1.42 (1.10-1.83) |
|  | Poultry only | 401 | 43 | -0.022 | 0.148 | 0.98 (0.73-1.31) | |  | | 200 | 244 | 0.047 | 0.053 | 1.05 (0.94-1.16) |
|  | Swine only | 308 | 136 | -0.066 | 0.106 | 0.94 (0.76-1.15) | |  | | 78 | 366 | 0.011 | 0.029 | 1.01 (0.95-1.07) |
| Acute URI | All | 3020 | 2797 | 0.030 | 0.038 | 1.03 (0.96-1.11) | |  | | 394 | 5423 | 0.031 | 0.012 | 1.03 (1.01-1.06) |
|  | Cattle only | 5603 | 214 | -0.023 | 0.107 | 0.98 (0.79-1.21) | |  | | 3805 | 2012 | 0.065 | 0.072 | 1.07 (0.93-1.23) |
|  | Goat only | 5078 | 739 | -0.056 | 0.160 | 0.95 (0.69-1.29) | |  | | 4087 | 1730 | 0.312 | 0.1000 | 1.37 (1.12-1.66) |
|  | Poultry only | 5300 | 517 | 0.027 | 0.090 | 1.03 (0.86-1.23) | |  | | 2321 | 3496 | 0.048 | 0.036 | 1.05 (0.98-1.12) |
|  | Swine only | 3704 | 2113 | 0.096 | 0.060 | 1.10 (0.98-1.24) | |  | | 739 | 5078 | 0.040 | 0.018 | 1.04 (1.01-1.08) |
| Pneumonia | All | 685 | 717 | 0.027 | 0.038 | 1.03 (0.95-1.11) | |  | | 119 | 1283 | 0.002 | 0.012 | 1.00 (0.98-1.03) |
|  | Cattle only | 1349 | 53 | -0.063 | 0.109 | 0.94 (0.76-1.16) | |  | | 1037 | 365 | 0.004 | 0.067 | 1.00 (0.88-1.15) |
|  | Goat only | 1155 | 247 | 0.344 | 0.136 | 1.41 (1.08-1.84) | |  | | 946 | 456 | 0.155 | 0.081 | 1.17 (1.00-1.37) |
|  | Poultry only | 1308 | 94 | -0.041 | 0.088 | 0.96 (0.81-1.14) | |  | | 569 | 833 | 0.006 | 0.032 | 1.01 (0.94-1.07) |
|  | Swine only | 884 | 518 | 0.051 | 0.059 | 1.05 (0.94-1.18) | |  | | 208 | 1194 | 0.000 | 0.017 | 1.00 (0.97-1.03) |
| Asthma | All | 1642 | 1327 | 0.026 | 0.026 | 1.03 (0.98-1.08) | |  | | 292 | 2677 | -0.003 | 0.008 | 1.00 (0.98-1.01) |
|  | Cattle only | 2820 | 149 | 0.089 | 0.079 | 1.09 (0.94-1.28) | |  | | 2129 | 840 | 0.062 | 0.053 | 1.06 (0.96-1.18) |
|  | Goat only | 2614 | 355 | 0.146 | 0.107 | 1.16 (0.94-1.43) | |  | | 2070 | 899 | 0.140 | 0.068 | 1.15 (1.01-1.31) |
|  | Poultry only | 2716 | 253 | 0.062 | 0.063 | 1.06 (0.94-1.20) | |  | | 1227 | 1742 | -0.014 | 0.024 | 0.99 (0.94-1.03) |
|  | Swine only | 2035 | 934 | 0.014 | 0.038 | 1.01 (0.94-1.09) | |  | | 535 | 2434 | -0.012 | 0.012 | 0.99 (0.96-1.01) |
| Aged 0 – 4 yrs | All | 106 | 89 | -0.198 | 0.113 | 0.82 (0.66-1.02) | |  | | 19 | 176 | 0.020 | 0.029 | 1.02 (0.96-1.08) |
| Aged 0 – 4 yrs | Cattle only | 188 | 7 | -0.028 | 0.285 | 0.97 (0.56-1.70) | |  | | 137 | 58 | 0.145 | 0.166 | 1.16 (0.83-1.60) |
| Aged 0 – 4 yrs | Goat only | 171 | 24 | -0.084 | 0.366 | 0.92 (0.45-1.88) | |  | | 114 | 81 | 0.482 | 0.175 | 1.62 (1.15-2.28) |
| Aged 0 – 4 yrs | Poultry only | 183 | 12 | -0.331 | 0.287 | 0.72 (0.41-1.26) | |  | | 104 | 91 | -0.062 | 0.082 | 0.94 (0.80-1.10) |
| Aged 0 – 4 yrs | Swine only | 134 | 61 | -0.240 | 0.182 | 0.79 (0.55-1.13) | |  | | 22 | 173 | 0.032 | 0.044 | 1.03 (0.95-1.13) |
| Aged 0 – 14 yrs | All | 315 | 298 | -0.033 | 0.054 | 0.97 (0.87-1.08) | |  | | 67 | 546 | -0.005 | 0.015 | 0.99 (0.97-1.03) |
| Aged 0 – 14 yrs | Cattle only | 584 | 29 | -0.117 | 0.146 | 0.89 (0.67-1.18) | |  | | 447 | 166 | -0.100 | 0.085 | 0.91 (0.77-1.07) |
| Aged 0 – 14 yrs | Goat only | 518 | 95 | 0.334 | 0.192 | 1.40 (0.96-2.04) | |  | | 404 | 209 | 0.316 | 0.099 | 1.37 (1.13-1.67) |
| Aged 0 – 14 yrs | Poultry only | 563 | 50 | -0.081 | 0.134 | 0.92 (0.71-1.20) | |  | | 299 | 314 | -0.076 | 0.043 | 0.93 (0.85-1.01) |
| Aged 0 – 14 yrs | Swine only | 418 | 195 | -0.043 | 0.084 | 0.96 (0.81-1.13) | |  | | 95 | 518 | 0.000 | 0.023 | 1.00 (0.96-1.05) |
| Hay fever | All | 1405 | 1176 | 0.006 | 0.033 | 1.01 (0.94-1.07) | |  | | 240 | 2341 | 0.001 | 0.011 | 1.00 (0.98-1.02) |
|  | Cattle only | 2442 | 139 | 0.074 | 0.093 | 1.08 (0.90-1.29) | |  | | 1860 | 721 | -0.014 | 0.065 | 0.99 (0.87-1.12) |
|  | Goat only | 2245 | 336 | 0.172 | 0.139 | 1.19 (0.90-1.56) | |  | | 1778 | 803 | 0.195 | 0.076 | 1.22 (1.05-1.41) |
|  | Poultry only | 2351 | 230 | 0.039 | 0.081 | 1.04 (0.89-1.22) | |  | | 1117 | 1464 | -0.019 | 0.030 | 0.98 (0.92-1.04) |
|  | Swine only | 1751 | 830 | -0.028 | 0.047 | 0.97 (0.89-1.07) | |  | | 414 | 2167 | -0.001 | 0.016 | 1.00 (0.97-1.03) |
| COPD | All | 948 | 886 | 0.040 | 0.037 | 1.04 (0.97-1.12) | |  | | 193 | 1641 | 0.002 | 0.010 | 1.00 (0.98-1.02) |
|  | Cattle only | 1746 | 88 | 0.078 | 0.101 | 1.08 (0.89-1.32) | |  | | 1281 | 553 | 0.046 | 0.062 | 1.05 (0.93-1.18) |
|  | Goat only | 1581 | 253 | 0.124 | 0.127 | 1.13 (0.88-1.45) | |  | | 1339 | 495 | 0.094 | 0.079 | 1.10 (0.94-1.28) |
|  | Poultry only | 1690 | 144 | 0.009 | 0.092 | 1.01 (0.84-1.21) | |  | | 733 | 1101 | 0.006 | 0.031 | 1.01 (0.95-1.07) |
|  | Swine only | 1181 | 653 | 0.054 | 0.057 | 1.06 (0.94-1.18) | |  | | 325 | 1509 | -0.004 | 0.015 | 1.00 (0.97-1.03) |
| Aged ≥ 45 yrs | All | 910 | 843 | 0.069 | 0.032 | 1.07 (1.01-1.14) | |  | | 184 | 1569 | -0.001 | 0.010 | 1.00 (0.98-1.02) |
| Aged ≥ 45 yrs | Cattle only | 1676 | 77 | 0.117 | 0.092 | 1.12 (0.94-1.35) | |  | | 1227 | 526 | 0.055 | 0.059 | 1.06 (0.94-1.19) |
| Aged ≥ 45 yrs | Goat only | 1508 | 245 | 0.143 | 0.120 | 1.15 (0.91-1.46) | |  | | 1280 | 473 | 0.019 | 0.077 | 1.02 (0.88-1.19) |
| Aged ≥ 45 yrs | Poultry only | 1617 | 136 | 0.097 | 0.074 | 1.10 (0.95-1.27) | |  | | 698 | 1055 | 0.003 | 0.028 | 1.00 (0.95-1.06) |
| Aged ≥ 45 yrs | Swine only | 1131 | 622 | 0.063 | 0.050 | 1.07 (0.97-1.17) | |  | | 311 | 1442 | -0.011 | 0.014 | 0.99 (0.96-1.02) |
| Aged ≥ 60 yrs | All | 716 | 647 | 0.120 | 0.049 | 1.13 (1.02-1.24) | |  | | 138 | 1225 | -0.025 | 0.015 | 0.98 (0.95-1.00) |
| Aged ≥ 60 yrs | Cattle only | 1303 | 60 | 0.143 | 0.151 | 1.15 (0.86-1.55) | |  | | 953 | 410 | 0.033 | 0.102 | 1.03 (0.85-1.26) |
| Aged ≥ 60 yrs | Goat only | 1169 | 194 | 0.223 | 0.221 | 1.25 (0.81-1.93) | |  | | 983 | 380 | -0.083 | 0.129 | 0.92 (0.71-1.19) |
| Aged ≥ 60 yrs | Poultry only | 1270 | 93 | 0.175 | 0.115 | 1.19 (0.95-1.49) | |  | | 548 | 815 | -0.035 | 0.047 | 0.97 (0.88-1.06) |
| Aged ≥ 60 yrs | Swine only | 883 | 480 | 0.127 | 0.079 | 1.14 (0.97-1.33) | |  | | 237 | 1126 | -0.045 | 0.024 | 0.96 (0.91-1.00) |
| Atopic eczema | All | 976 | 899 | 0.022 | 0.028 | 1.02 (0.97-1.08) | |  | | 154 | 1721 | 0.012 | 0.009 | 1.01 (0.99-1.03) |
|  | Cattle only | 1764 | 111 | 0.090 | 0.079 | 1.09 (0.94-1.28) | |  | | 1314 | 561 | 0.093 | 0.055 | 1.10 (0.99-1.22) |
|  | Goat only | 1588 | 287 | 0.233 | 0.117 | 1.26 (1.00-1.59) | |  | | 1343 | 532 | 0.175 | 0.076 | 1.19 (1.03-1.38) |
|  | Poultry only | 1678 | 197 | -0.042 | 0.063 | 0.96 (0.85-1.09) | |  | | 711 | 1164 | 0.051 | 0.026 | 1.05 (1.00-1.11) |
|  | Swine only | 1240 | 635 | 0.023 | 0.045 | 1.02 (0.94-1.12) | |  | | 257 | 1618 | 0.002 | 0.013 | 1.00 (0.98-1.03) |
| Aged 0 – 4 yrs | All | 245 | 246 | 0.091 | 0.048 | 1.10 (1.00-1.20) | |  | | 44 | 447 | -0.019 | 0.014 | 0.98 (0.96-1.01) |
| Aged 0 – 4 yrs | Cattle only | 464 | 27 | 0.134 | 0.129 | 1.14 (0.89-1.47) | |  | | 346 | 145 | -0.047 | 0.080 | 0.95 (0.82-1.12) |
| Aged 0 – 4 yrs | Goat only | 413 | 78 | 0.333 | 0.180 | 1.40 (0.98-1.99) | |  | | 363 | 128 | 0.029 | 0.108 | 1.03 (0.83-1.27) |
| Aged 0 – 4 yrs | Poultry only | 436 | 55 | 0.052 | 0.116 | 1.05 (0.84-1.32) | |  | | 199 | 292 | -0.029 | 0.041 | 0.97 (0.90-1.05) |
| Aged 0 – 4 yrs | Swine only | 313 | 178 | 0.092 | 0.075 | 1.10 (0.95-1.27) | |  | | 60 | 431 | -0.032 | 0.021 | 0.97 (0.93-1.01) |
| Aged 0 – 14 yrs | All | 420 | 410 | 0.057 | 0.040 | 1.06 (0.98-1.15) | |  | | 72 | 758 | -0.005 | 0.012 | 0.99 (0.97-1.02) |
| Aged 0 – 14 yrs | Cattle only | 775 | 55 | 0.164 | 0.103 | 1.18 (0.96-1.44) | |  | | 584 | 246 | 0.029 | 0.069 | 1.03 (0.90-1.18) |
| Aged 0 – 14 yrs | Goat only | 702 | 128 | 0.380 | 0.151 | 1.46 (1.09-1.97) | |  | | 608 | 222 | 0.026 | 0.093 | 1.03 (0.86-1.23) |
| Aged 0 – 14 yrs | Poultry only | 741 | 89 | -0.057 | 0.098 | 0.94 (0.78-1.15) | |  | | 316 | 514 | 0.023 | 0.036 | 1.02 (0.95-1.10) |
| Aged 0 – 14 yrs | Swine only | 538 | 292 | 0.041 | 0.063 | 1.04 (0.92-1.18) | |  | | 104 | 726 | -0.016 | 0.018 | 0.98 (0.95-1.02) |

*Note.* CAFO = concentrated animal feeding operations; OR =odds ratio; CI = confidence interval; URI = upper respiratory infection.

^a^ For each additional CAFO; adjusted for age, gender, registry duration, the number of inhabitants in the postal code area and total surface area.

^b^ Defined as >250 dairy cows or >2,500 veal calves (cattle CAFO), >7,500 finishing pigs or >1,200 breeding sows (swine CAFO), >120,000 laying hens or >220,000 broilers (poultry CAFO), or >1,500 goats (goat CAFO).
